# A systematic framework for predictive biomarkers in immune effector cell-associated neurotoxicity syndrome

**DOI:** 10.3389/fneur.2023.1110647

**Published:** 2023-02-10

**Authors:** Omar H. Butt, Alice Y. Zhou, Beau M. Ances, John F. DiPersio, Armin Ghobadi

**Affiliations:** ^1^Division of Oncology, Department of Medicine, Siteman Cancer Center, Washington University in Saint Louis, St. Louis, MO, United States; ^2^Department of Neurology, Washington University in Saint Louis, St. Louis, MO, United States

**Keywords:** ICANS, neurotoxicity, predictive biomarker, review, cellular therapies, CRS, cytokine release syndrome, cytokines

## Abstract

Chimeric antigen receptor (CAR)-T cell therapy has revolutionized the management of several life-threatening malignancies, often achieving durable sustained responses. The number of patients treated with this new class of cell-based therapy, along with the number of Food and Drug Association (FDA) approved indications, are growing significantly. Unfortunately Immune Effector Cell-Associated Neurotoxicity Syndrome (ICANS) can often occur after treatment with CAR-T cells, and severe ICANS can be associated with significant morbidity and mortality. Current standard treatments are mainly steroids and supportive care, highlighting the need for early identification. In the last several years, a range of predictive biomarkers have been proposed to distinguish patients at increased risk for developing ICANS. In this review, we discuss a systematic framework to organize potential predictive biomarkers that builds on our current understanding of ICANS.

## Introduction

Development of cellular therapies has expanded rapidly since the FDA approval of the first commercial cellular therapy, tisagenlecleucel, in 2017. As of the time of this review, there are six FDA-approved products with nearly 3,000 clinical trials focused on experimental cellular therapies under active investigation, representing a 50% growth from the prior year (1). Of these, T cell based therapies remain the most common (52%) with a majority targeting CD19 (56%). The number of patients treated with cellular therapies also continues to increase with approximately 5,000 annually receiving CAR-T in the US (1). Unfortunately, between 15 and 65% of treated patients developed Immune Effector Cell-Associated Neurotoxicity Syndrome (ICANS) in the original randomized control trials (RTC) for the six approved agents (Table 1), and upwards of 55 to 69% has been reported in large real-world cohorts (16, 17).

**Table 1:** FDA-approved cellular therapy: frequency of neurotoxicity and related adverse events.

**Approved agent**	Indication^*^	**Approval year**	**Original trial**	**CRS frequency**	**Grade 3+ CRS frequency**	**CRS median onset day/duration**	**ICANS frequency**	**Grade 3+ ICANS frequency**	**Neuro-toxicity median onset day/duration**	**Other neuro-toxicity**	**Cerebral edema frequency**	**Ref**.
Tisagenlecleucel	B-ALL	2017	ELIANA	77	21	3/8	40	13	Not Reported	**	0	(2)
	DLBCL	2018	JULIET	58	22	3/7	21	12	6/14	-	0	(3)/ (4)
	FL	2022	ELARA	48.5	0	4/4	4.1	1	9/2 (reported for severe only)	37.1	0	(5)
Axicabtagene ciloleucel	DLBCL	2017	ZUMA-1	93	13	2/8	64	28	5/Not reported (median resolution Day 17)	-	-	(6)
	FL	2021	ZUMA-5	82 (78% in FL subgroup)	7 (6% in FL subgroup)	4/6 (FL subgroup)	59 (56% in FL subgroup)	19 (15% in FL subgroup)	7/14 (FL subgroup)	-	0	(7)
	LBCL (2^nd^ line)	2022	ZUMA-7	92	6	3/7	60	21	7/9	^†^	0	(8)
Brexucabtagene autoleucel	MCL	2020	ZUMA-2	91	15	2/11	63	31	7/12	-	1	(9)
	B-ALL	2021	ZUMA-3	89	24	5/7.5	60	25	9/7	-	1^††^	(10)
Lisocabtagene maraleucel	DLBCL	2021	TRANSEND NHL 001	42	2	5/5	30	10	9/11	^‡^	0	(11)
	DLBCL (2^nd^ line)	2022	TRANSFORM	49	1	5/4	12	4	11/6	-	0	(12)
Idecabtagene vicleucel	MM	2021	KarMMa	84	5	1/5	18	3	2/3	-	0	(13)
			KarMMa-3^ϕ^	85	9	1/7	28	4	2/5	-	1^‡‡^	(14)
Ciltacabtagene autoleucel	MM	2022	CARTITUDE-1	95	4	7/4	17	2	8/4 (27 day onset for nonICANS)	12	0	(15)

* Relapsed/refractory unless otherwise stated as second line.

** One patient died of cerebral hemorrhage and two patients developed grade 4 encephalitis, all determined by investigators to be unrelated to tisagenlecleucel.

† One patient died from PML, one from stroke, both determined by investigators to be unrelated to axicabtagene ciloleucel.

†† One patient developed grade 4 encephalopathy and 1 patient developed brain herniation attributed to brexucabtagene autoleucel.

‡ One instance of fludarabine-associated leukoencephalopathy determined by investigators to be unrelated to lisocabtagene maraleucel.

‡‡ Reported one case of cerebral edema in another study using same agent, idecabtagene vicleucel.

ϕ Interim data as of 8/10/22.

All indications are for relapsed/refractory disease unless otherwise stated (e.g., Axi-cel and Liso-cel recently obtained 2nd line indication). ZUMA-5 examined both FL and MZL, with the entire cohort as well as the FL subset included here. B-ALL, B-cell acute lymphoblastic leukemia; CRS, Cytokine release syndrome; DLBCL, Diffuse large B-cell lymphoma; FL, Follicular lymphoma; ICANS, Immune effector cell-associated neurotoxicity syndrome; MCL, Mantel cell lymphoma; MM, Multiple myeloma; MZL, Marginal zone lymphoma; PMID, PubMed Identifier Number.

Neurologic symptoms associated with ICANS includes mild confusion, inattention, impaired reading, disorientation, word-finding difficulty, delirium, and impaired consciousness. This wide range of symptoms can be clustered using American Society for Transplantation and Cellular Therapy (ASTCT) consensus grading (18). Low grade (grade 1–2) ICANS is characterized by mild loss of orientation, naming, commands, writing, or attention as assessed using the Immune-Effector Cell-Associated Encephalopathy (ICE) Tool (18). Most cases of grade 1 or 2 ICANS are self-limited and resolve with supportive care. The further presence of focal neurological weakness, seizure, depressed consciousness, define grade 3 ICANS and represent a medical emergency given the risk of rapid progression to grade 4 with associated coma, recurrent or prolonged seizures, or cerebral edema (18). Diffuse grade 4 cerebral edema remains a comparatively rare, but nonetheless a concerning risk for all agents (Table 1). Also notable are neurologic symptoms *not* included in current ICANS grading schema, including tremors, myoclonus, and intracranial hemorrhage. Early identification remains essential given the significant risk of morbidity and mortality associated with ICANS.

Treatment options remain limited; management is primarily through aggressive supportive care and high-dose steroids (19–21). As such, current management guidelines for ICANS stresses early identification by active monitoring and serial assessments. However, the optimal timing and dosing of steroids remains debated. Steroids are potent anti-inflammatory therapy that decrease immune cell proliferation, cytokine production, and cytotoxic activity, raising concern for impaired CAR-T efficacy. While some reports suggest no influence of steroids on CAR-T kinetics (22), other reports reveal cumulative dose and duration of steroids is associated with shorter progression-free and overall survival after cellular therapy (23). This has led to several ongoing early phase clinical trials aimed to identify targeted immunomodulatory or other steroid-sparing interventions that does not impair CAR-T cell efficacy. Given the significant risk associated with ICANS and limited treatment options to date, early identification remains essential.

## ICANS in FDA-approved cellular therapies

ICANS was originally observed during phase I/II trials of all current FDA-approved cellular therapies. Incidence varied between different cellular therapies and even for the same agent when used for different indications (Table 1). The highest rates of neurotoxicity were observed in the original ZUMA trials for axicabtagene ciloleuce [axi-cel, 78% or 330/422 aggregated unique total patients, including 64% or 65/111 (ZUMA-1), 59% or 87/148 (ZUMA-5), and 60% or 102/170 (ZUMA-7)] and for brexucabtagene autoleuce [brexu-cel, 63% or 43/74 (ZUMA-2)]. Tisagenlecleucel (tisa-cel) by comparison had a lower reported incidence at 40% or 30/75 (ELIANA trial) while Idecabtagene vicleucel (ide-cel) and ciltacabtagene autoleucel (cilta-cel) had the lowest incidence of neurotoxicity. Symptom onset mainly ranged between 5 and 11 days, though earlier (median day 2) ICANS was reported for ide-cel (KarMMa trial).

Comparing between different agents suggests a relationship between the incidence of neurotoxicity, a given target, and the costimulatory domain used in a given agent. The highest rates are observed in CD19-directed agents using containing a CD28 co-stimulatory domain (e.g., axi-cel and brexu-cel), with lower rates observes for CD19-directed agents using containing a 4-1BB co-stimulatory domain (tisa-cel and liso-cel). Ide-cel and cilta-cel both target B cell maturation antigen (BCMA) and also use a 4-1BB costimulatory domain. In contrast to their CD19-directed peers, ide-cel and cilta-cel have the lowest incidence of neurotoxicity (Table 1). While the selected agent remains a major risk factor for ICANS, it is only one of several factors associated with ICANS risk after cellular therapy.

## Predictive biomarkers for adverse events after cellular therapy

Predictive biomarkers aid in early diagnosis, optimal apportioning of clinical resources, early intervention including prophylactic or preemptive treatment for high risk patients, and patient risk stratification. Early success was observed for the closely related adverse event known as cytokine release syndrome (CRS) with efficacious targeted interventions for CRS now available. CRS is an exaggerated systemic inflammatory response which manifests within the first 96 h after treatment in between 40 and 90% of all patients (Table 1). Fever is the hallmark symptom with severe cases associated with hypotension requiring vasopressors, hypoxia requiring mechanical support, and end-organ damage (18). Intimately connected to early cytokine elevations, targeted intervention has ameliorated significant morbidity and mortality associated with severe CRS. Since ICANS was first observed, a similar concerted effort has been devoted to identify potential predictive biomarkers. Unfortunately predictive biomarkers for ICANS remained elusive until only recently, with significant gaps in mechanistic understanding.

Challenges in identifying and validating predictive biomarkers in ICANS are rooted in several factors: First, small multi-center or single center studies have limited sample size, and studies often lack power to infer relationships in the subgroup of patients with ICANS. Second, the incidence rate of ICANS varies widely between studies and agents, making it difficult to draw conclusions between studies. Third, patients who develop ICANS often (but not always) also have ongoing CRS, making it difficult to disentangling a putative ICANS specific biomarker. Fourth, it is unclear if risk factors for ICANS are different between adult and pediatric patients, confounded generalizability between groups. Finally, until recently there are no animal models of ICANS to study its pathophysiology and mechanism outside of retrospective correlative clinical studies. In the following sections we will review all major predictive biomarkers associated with neurotoxicity after cellular therapy while keeping these challenges in mind.

## Three major classes of predictive biomarker classes for ICANS

**Figure 1:**
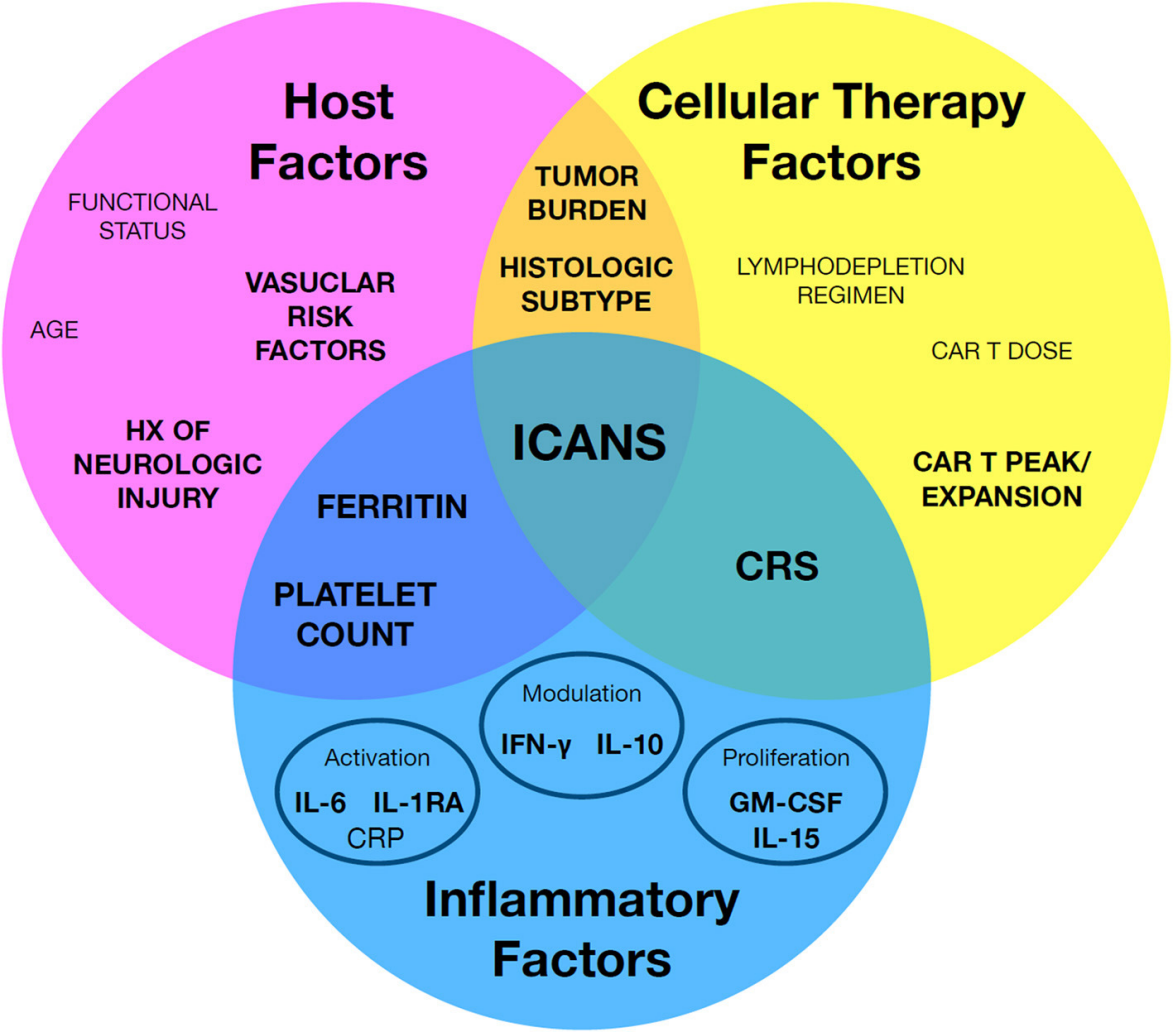
A framework to organize known immune effector cell-associated neurotoxicity syndrome (ICANS) risk factors. Individual factors may be broadly grouped by host factors, cellular therapy factors, and inflammatory factors. Notably, a subset of key factors bridge between classes, such as cytokine release syndrome (CRS). Factors with the strongest associated evidence are in highlighted in bold.

Predictive biomarkers for ICANS may be clustered in one of three major categories: (1) host factors which increase a given patient's risk for ICANS, (2) cellular therapy factors related to the cellular product, and (3) inflammatory factors relating to intersection of the first two categories, including most importantly CRS (Figure 1). The following sections will review reported predictive biomarkers of ICANS risk and discuss caveats with the interpretation and application of these biomarkers.

## Host factors

Commonly reported host factors that can affect the development of ICANS include age, functional status, tumor burden, histologic subtype of tumor, neurologic comorbidities, and vascular risk factors.

### Age

Age has been suggested as a risk factor for the development of ICANS (Table 2). However, evidence is conflicting and strongly biased by agent examined: The original ZUMA-1 clinical trial which lead to approval for axi-cel for DLBCL reported modest increased risk of neurologic events in patients ≥65 years old (*n* = 27, median age 69) vs. <65 years of age (*n* = 81, median age 55). Grade 3+ neurologic toxicities included delirium (11 vs. 0%), agitation (11 vs. 2%), and encephalopathy (30 vs. 22%), but interestingly not aphasia (0 vs. 10%) (50). Rubin et al. prospectively examined a cohort of 204 patients receiving axi-cel (median age 60 years) and found 58% developed neurotoxicity, with 43% developing grade 3+ neurotoxicity (39). Univariate regression revealed an association between age and ICANS (odds ratio (OR) 1.05). Once axi-cel was FDA approved for use, Jacobson et al. examined outcomes in a large international cohort of 1,297 patients who received the standard of care axi-cel, of which 739 (57%) would have been illegible for the original ZUMA-1 clinical trial (16) due to age or other ineglibility criteria. In the clinical practice, 55% of patients developed ICANS, with 24% developing grade 3+ ICANS. Multivariate analysis revealed a significant increased risk for patients aged 65 and older of developing any grade ICANS and grade 3+ ICANS with an odds ratio (OR) of 1.77 (95% CI, 1.39 to 2.26) and 1.38 (95% CI, 1.04–1.82) respectively. However, this relationship between older age and ICANS in patients treated with axi-cel has not been universally observed. Faramand and colleagues prospectively examined 75 patients (median age 63) treated with axi-cel and found no association with age. Similarly, Nastoupil and colleagues retrospectively analyzed results from the US Lymphoma CAR-T consortium totally 298 patients (median age 60, of whom, 275 were treated with axi-cel) and found no association with age and severe (Grade 3) neurotoxicity. No association with age has likewise been reported in smaller retrospective cohorts as well (40, 43, 48, 51). Like axi-cel, the investigational agent 19–28 z uses a CD28 co-stimulatory domain. Park et al. initially reported an association with age and ICANS in 51 adult patients (30), although this relationship was not observed in a second adult cohort of 51 patients (32). Overall, conflicting evidence exists for axi-cel and similar CD19-directed agents.

**Table 2:** Summary of predictive and associated neurotoxicity markers by study.

**Study**	***n***	**Age**	**Agent**	**Construct**	**Host factors**	**Cellular therapy factors**	**Inflammatory + post-infusion factors**
Turtle et al. (24)	32 (29 evaluable)	40	Investigational (JCAR014)	FMC63 CD19-CD28-4-1BB-CD3ζ	Tumor burden	CAR T cell dose LD regimen	CRS D1 TNF D1+Peak IL-6 D1+Peak IFN-γ Peak ferritin Peak CRP
Turtle et al. (25)	37	57	Investigational (JCAR014)	FMC63 CD19-CD28-4-1BB- CD3ζ	-	CAR T cell dose CAR T expansion/peak LD regimen	D1 + Peak IL-6 D1 + Peak IFN-γ D1 + Peak IL-15 D1 IL-8 D1 IL-10 Peak IL-2 Peak IL-18 Peak TIM-3 Peak ferritin Peak CRP D1 + Nadir TGF-β
Gardner et al. (26)	45	12.2	Investigational (SCRI-CAR19v1)	CD19-CD3ζ/CD28	NOT Tumor burden	NOT dose level NOT LD regimen/dose	CRS
Turtle et al. (27)	24	61	Investigational (JCAR014)	FMC63 CD19-CD28-4-1BB- CD3ζ	-	CAR T expansion/peak	CRS D2 + Peak IFN-γ D2 + Peak IL-10 Peak IL-6 Peak MCP1 Peak TNFRp55 Peak sIL2Rα Peak sIL6R Peak TIM3 Peak CRP Peak Ferritin
Gust et al. (28)	133	Between 40 and 60	Investigational (JCAR014)	FMC63 CD19-CD28-4-1BB- CD3ζ	Tumor burden Neurologic comorbidities 36hr + nadir Platelets High ANG2 High ANG2/ANG1 High VWF D9 fibrinogen (drop)	CAR T cell dose LD regimen/dose CAR T expansion/peak	CRS 36 h MCP-1 36 h IL-15 36 h IL-10 36 h IL-2 36 h + Peak IL-6 36 h + Peak IFN-γ 36 h + Peak CRP D2 + Peak ferritin D2 + Peak D-dimer CSF IFN-γ CSF TNF CSF IL6
Kochenderfer et al. (29)	22	53.5	Investigational (CD-19)	CD19-CD-28//CD3ζ	-	CAR T expansion/peak	Peak IL-10 Peak IL-15 Peak GranzymeA Peak GranzymeB Peak PLGF
Park et al. (30)	51	Not reported	Investigational (19–28 z)	CD19-CD3ζ/CD28	NOT age tumor burden	NOT T cell dose NOT LD regimen CAR T expansion/peak	CRS D3 IL-2 D3 IL-5 D3 LL-10 D3 IL-15 D3 GM-CSF D3 IFNγ Peak Ferritin
Neelapu et al. (6)	111	58	Axi-cel	CD19-CD28-CD28/CD3ζ	-	CAR T expansion/peak	Peak IL-1RA Peak IL-2 Peak IL-6 Peak IL-8 Peak IL-10 Peak IL-15 Peak GM-CSF Peak IFN-γ Peak CCL2 Peak Peak Ferritin Peak GranzymeB
Gofshteyn et al. (31)	51	11.5	Tasi-cel	CD19-CD8α-4-1BB/CD3ζ	History of pre-existing neurologic deficit	-	CRS D3 sTNFR-1 Peak IL-2 Peak sIL-4R Peak HGF Peak IL-15
Santomasso et al. (32)	53	Not Reported	Investigational (19–28 z)	CD19-CD3ζ/CD28	Age Tumor burden Low Platelets Low fibrinogen Low ANG1 High ANG2	CAR T expansion/peak	Baseline + D3 + Peak IL-10 Baseline + D3 + Peak GM-CSF Baseline + D3 + Peak G-CSF Baseline + Peak IP10 Baseline + D3 + Peak IL-6 CRS D2 + Peak CRP D4 + Peak Ferritin D3 + Peak IL-1α D3 + Peak IL-2 D3 + Peak IL-3 D3 + Peak IL-5 D3 + Peak IL-15 Peak fractalkine D3 + Peak MCP-1 D3 + Peak IFNγ CSF Protein CSF/Serum Albumin Ratio CSF IL1α +IL6+IL10+G-CSF+TNF+IFNγ+IFNα2+FLT3L+eotaxin
Maude et al. (2)	75	11	Tasi-cel	CD19-CD8α-4-1BB/CD3ζ	-	-	CRS
Shalabi et al. (33)	22	17.9	Investigational (Anti-CD22)	CD-22-4-1BB	Three of five patients w history of pre-existing neurologic deficits developed ICANS	-	Peak IL-2 Peak IL-6 Peak IL-8 Peak IL-10 Peak IL-13 Peak IL-15 Peak TNF Peak GM-CSF
Cohen et al. (34)	25	58	Investigational (BCMA)	BCMA-4-1BB	High tumor burden	-	CRS Peak IFN-γ Peak IL-1RA Peak MIP-1α Peak IL-1β Peak IL-2Rα Peak IL-6 Peak IL-7 Peak IL-10 Peak IL-15 Peak GM-CSF
Curran et al. (35)	25	13.5	Investigational (19–28 z)	CD19-CD3ζ/CD28	NOT disease burden	NOT CAR T dose NOT CAR T peak NOT LD regimen/dose	CRS
Gust et al. (36)	43	12.5	Investigational (SCRI-CAR19v1)	CD19-CD3ζ/CD28	Race Abnormal MRI NOT Age NOT pre-existing neurologic conditions NOT tumor burden NO Ang-2/Ang-1 ratio NO nadir fibrinogen	CAR T expansion/peak NOT CART T dose NOT LD regimen	CRS CSF Peak IFN-γ CSF Peak IL-6 CSF Peak GranzymeB CSF Peak protein CSF Peak WBC CSF Peak GFAP CSF Peak S100b D7 IFN-γ D7 IL-10 D7 GranzymeB
Karschnia et al. (37)	25	Not reported	Multiple (24/25 CD-19 directed, 1/25 α-fetoprotein directed)	Multiple	NOT age Nadir Platelets, NOT baseline	-	CRS Peak ferritin NOT CRP
Faramand et al. (38)	75	63	Axi-cel	CD19-CD28-CD28/CD3ζ	NOT Age NOT ECOG Low ANG1 High ANG2 High ANG2/ANG1	-	Baseline + Peak IL6 Baseline ferritin D0 IL-15 Peak IFN-γ
Abramson et al. (11)	269	63	Liso-cel	CD19-CD28-4-1BB/CD3ζ	-	CAR T expansion/peak	-
Rubin et al. (39)	204	60	Axi-cel	CD19-CD28-CD28/CD3ζ	Age Histological subtype	-	CRS Fever Peak CRP Peak ferritin Peak WBC Peak IL-6
Nastoupil et al. (17)	298	60	Axi-cel	CD19-CD28-CD28/CD3ζ	NOT age High tumor burden LEVF < 50% NOT subtype	-	NOT LDH
Wang et al. (9)	68	65	Brexu-cel	CD19-CD28-CD28/CD3ζ	-	CAR T expansion/peak	Peak IL-1RA Peak IL-2 Peak IL-6 Peak IL-10 Peak IL-12p40 Peak TNF Peak GM-CSF Peak IFN-γ Peak CCL13 Peak CCL4 Peak GranzymeB Peak CSF CRP Peak CSF IL-6 Peak CSF IL-8 Peak CSF VCAM-1
Schuster et al. (4)	115	56	Tasi-cel	CD19-CD8α-4-1BB/CD3ζ	-	-	Pre-infusion CRP CRS D1+ CRP D6+ Ferritin
Shah et al. (10)	55	40	Axi-cel	CD19-CD28-CD28/CD3ζ	-	CAR T expansion/peak	Baseline GranzymeB Baseline IL-8 Peak IL-1RA Peak IL-6 No difference in ferritin, GM-CSF, IL15
Wudhikarn et al. (40)	78	58.8	Axi-cel	CD19-CD28-CD28/CD3ζ	ECOG ≥2 (< 60 years old only)	-	CRS
Jacobson et al. (7)	148	61	Axi-cel	CD19- CD28-CD28/CD3ζ	-	CAR T expansion/peak	Baseline Ferritin* Peak IL-2* Peak IL-4* Peak IL-5* Peak IL-6* Peak IL-10* Peak IL-12* Peak IL-15* Peak IFN-γ^*^ Peak GM-CSF* Peak CCL2* Peak GranzymeB* Peak TNF* ^*^for follicular lymphoma subset only
Jacobson et al. (16)	1,297	63.1	Axi-cel	CD19-CD28-CD28/CD3ζ	Age ECOG ≥2	-	
Riedell et al. (41)	240	59 (axi-cel)/67 (tisa-cel)	Axi-cel/Tasi-cel	Multiple agents	ECOG ≥2 Hx of ≥4 prior lines of therapy	-	Peak ferritin ≥5,000
Iacoboni et al. (42)	75	60	Tasi-cel	CD19- CD8α-4-1BB/CD3ζ	ECOG ≥1 Refractory disease	CART T dose	Baseline LDH
Gauthier et al. (43)	129	62 (axi-cel)/64 (tisa-cel)/60 (JCAR014)	Axi-cel/Tasi-cel/JCAR014	Multiple agents	NOT Age NOT tumor burden	-	NOT pre-treatment LDH, NOT pre- treatment ALC, NOT LDH
Möhn et al. (44)	15	59	Tasi-cel	CD19-CD8α-4-1BB/CD3ζ	Pre-existing neurologic conditions	CART T dose	CRS
Schoeberl et al. (45)	96	58	Axi-cel, Tasi-cel, investigational	Multiple agents	Baseline serum NfL	-	CRS Peak NfL* *whole cohort, ICANS subgroup *not reported*
Butt et al. (46)	30	64	Axi-cel, Tasi-cel, investigational	Multiple agents	Baseline plasma NfL	-	CRS Baseline ferritin NOT peak NfL
Qi et al. (47)	48	31	Investigational (multiple)	Multiple agents	Active CNS disease	CAR T expansion/peak	CRS Peak IL-6 Peak CSF blasts
Tang et al. (48)	77	Between 58–66	Axi-cel or Tasi-cel	Multiple agents	NOT Age	-	CRS Peak CRP Phosphate < 2 Nadir phosphate
Gust et al. (49)	141	12.2	Investigational (multiple)	Multiple agents	NOT Preexisting neurologic comorbidities NOT baseline serum NfL or GFAP	-	CRS CSF GFAP change CSF NfL change (CD-19 CAR T subset only)

Study trial size, median age, and agent(s) including construct is included for reference. Key randomized clinical trials associated with drug FDA-approval (see Table 1) are colored in gray background.

A relationship between age and ICANS was not observed for tisa-cel, either in the original trials (3) or later real-world cohorts (42, 43). Recently *younger*, not older age was reported associated with ICANS in a cohort of 15 patients (median age 59) treated with tisa-cel (44) (Table 2). Given the comparatively low incidence of ICANS for tisa-cel compared to axi-cel, statistical power is a limiting factor in drawing conclusions on the risk factors for ICANS development with tisa-cel. When examining an investigational CD19-directed CAR-T that also contains a 4-1BB costimulatory domain like tisa-cel, Gust et al. (28) also did not observe a significant relationship between neurotoxicity and age, but interestingly a trend for increased ICANS in *younger*, not older adult patients. Overall, age is an unclear risk factor with differences predominately related to agent. Larger studies are needed to understand the contributing factor of age to ICANS.

### Functional status

Functional status, like age, is a risk factor reported predominately in studies that are heavily biased toward CD19-directed agents. It is measured using Eastern Cooperative Oncology Group performance status (ECOG PS), an established ordinal scale capturing a patient's level of medical independence and activity. An ECOG PS of 0 reflects a fully independent patient, while ECOG PS of 2 reflects an ambulatory patient who can carry out self-care but unable to work and up no more than 50% of waking hours. Faramand et al. (38) prospective study of patients treated with axi-cell did not observe an association between ECOG score and ICANS. In contrast, several large retrospective studies have demonstrated a positive association: Riedell and colleagues performed a retrospective multi-center study on 240 patients undergoing cellular therapy for a B cell lymphoma. They also observed ECOG PS ≥2 was associated with an increased risk of developing ICANS (*p* = 0.05, OR 5.05), even when controlling for treatment type i.e., axi-cel use. Jacobson and colleagues real-world cohort of 1,297 patients treated with axi-cel also observed a significant OR for baseline ECOG PS ≥2 and both any grade ICANS (OR 2.63; 95% CI, 1.40 to 4.93) and grade 3+ ICANS (3.23; 95% CI, 1.81 to 5.74) (16). Similarly, Iacaboni et al. examined 75 patients (median age 60) treated exclusively with tisa-cel in a retrospective Spanish cohort and found an association between ICANS and ECOG FS ≥1 (vs. 0) (42). Wudhikarn et al. only observed an increased likelihood of developing ICANS in patients with ECOG ≥2, but only in patients under the age of 60 (40). Gauthier and colleagues did not observe an association in their smaller retrospectively study (43). Taken together, ECOG PS remains a possible but weak factor mainly observed in larger cohorts with limited data beyond CD19-directed agents.

### Tumor burden

Pre-infusion tumor burden has been reported as a risk factor for the development of neurotoxicity across several (17, 24, 28, 30, 32, 34) but not all studies (26, 36, 43, 44). Notably the methods used to measure tumor burden can vary widely from bone marrow assessments, to imaging-derived (including using positron emission tomography (PET), discussed below), to secondarily inferred from blood LDH levels (Table 3). As a result, tumor burden has been defined in different studies as a categorical variable (“high” vs. “low burden” based on marrow blasts or by the presence of secondary sites of disease), ordinal variable, and continuous variable highlighting the varied approaches used. To date, the association between tumor and risk for ICANS has been best characterized in adults with B cell lymphomas (17, 24, 28, 30, 32, 52) but not children (36). Studies reporting no association often limited by power (43, 44). Taken together, pre-infusion tumor burden is a possible risk factor, though standardization of measurement modality and larger studies comparing across agents is warranted.

**Table 3:** Summary of studies reporting tumor burden including modality of measurement and reported relationship with neurotoxicity.

**Study**	**Agent**	**Study population**	**Measurement**	**Relationship**
Turtle et al. (24)	Investigational (JCAR014)	Adult B-ALL	Bone Marrow % Blasts	Higher associated with CRS, CAR-T expansion, neurotoxicity
Gust et al. (28)	Investigational (JCAR014)	Adult B-ALL, NHL, CLL	% Total CD19+ cells in marrow	Uni+Multivariate association along with pre-existing neurologic comorbidities, increased CAR-T expansion, lymphodepletion regimen, infused CAR-T dose
Park et al. (30)	Investigational (19–28 z)	Adult B-ALL	% Blasts	Uni+Multivariate association along with low baseline platelet count (< 60) and >5% blasts
Santomasso et al. (32)	Investigational (19–28 z)	Adult B-ALL	High disease burden = blasts ≥5% or extra-medullary disease on imaging; Low disease burden = < 5% bone marrow blast	High disease burden associated with neurotoxicity using a Fisher exact test
Cohen et al. (34)	Investigational (BCMA)	Adult MM	Bone Marrow % plasma cells	All 3 subjects with grade 3+ neurotoxicity had high tumor burden (2 of 3 with extramedullary disease), received a high dose of CART-BCMA cells, and had grade 3+ CRS
Nastoupil et al. (17)	Axi-cel	Adult LBCL	Bulky disease ≥ 10 cm	Uni+Multivariate association, although not associated with age, CNS involvement, lymphoma-subtype
Gardner et al. (26)	Investigational (SCRI-CAR19v1)	Pediatric B-ALL	CD19+ cells in marrow grouped by MRD	NO association with tumor burden (as defined) was observed
Gust et al. (36)	Investigational (SCRI-CAR19v1)	Pediatric B-ALL	% Total CD19+ cells in marrow	NO association with tumor burden (as defined) was observed
Gauthier et al. (43)	Axi-cel/Tasi-cel/JCAR014	Adult B-NHL	Bulk (largest lesion diameter) and inferred from LDH	NO association with tumor burden (as defined) was observed
Möhn et al. (44)	Tasi-cel	Adult LBCL	Inferred from baseline LDH	No significant association observed

ALL, Acute lymphoblastic leukemia; B-ALL, B-cell acute lymphoblastic leukemia; CLL, Chronic lymphocytic leukemia; CNS, Central nervous system; LBCL, Large B-cell lymphoma; LDH, Lactate dehydrogenase; MRD, Minimal residual disease; NHL, Non-Hogkin's lymphoma.

PET-derived assessment of total metabolic tumor volume (TMTV) has been proposed as a standardized means to quantify total active tumor volume and address some of the variability in tumor burden assessment highlighted above. TMTV is calculated from pre-treatment (i.e., baseline at recurrence) PET scans of patients with B cell lymphoma using a 41% maximum standardized uptake value threshold (53, 54). Unfortunately use of TMTV has had mixed to success to date (52, 54). Dean et al. retrospectively examined baseline PET scans of 96 patients treated with axi-cel and found no relationship between TMTV and ICANS (54), in line with earlier reports (55). Likewise, Iacoboni et al. retrospectively examined a cohort of 35 patients treated with an admixture of agents (breakdown not reported) also did not observe an association with ICANS risk (52). Nor did we see a relationship between TMTV and ICANS risk in our recent retrospective study of 30 patients (46). TMTV as a surrogate for tumor burden in B cell lymphomas remains of unclear utility at this time.

### Histologic subtype of tumor

Until recently, only axi-cel's multiple indications permitted comparison of ICANS incidence between different histological subtypes while controlling for agent. The original ZUMA-1 [diffused large B cell lymphoma (DLBCL)], ZUMA-5 [follicular lymphoma (FL)], and most recently ZUMA-7 (2nd line for large B cell) clinical trials all reported comparable frequency of ICANS of approximately 60% (Table 1). However, the frequency of grade 3+ ICANS was approximately half for FL (ZUMA-5) vs. DLBCL (ZUMA-1). Rubin and colleagues prospective study of 204 patients categorically clustered by patient's histological subtype: aggressive (DLBL, primary mediastinal B-cell lymphoma) or indolent (follicular lymphoma, marginal zone lymphoma) histologic subtype (39). They found a univariate association between neurotoxicity and an aggressive histological subtype (*p* < 0.001), and included it in their multivariate prediction model. When comparing between different aggressive subtypes of lymphoma (DLBL, primary mediastinal B-cell lymphoma, transformed FL, germinal center B cell (GCB)-like, non-GCB, double/triple hit, or double expressor), no association was observed retrospectively in the US Lymphoma CAR-T consortium (17). With the recent approval of tisa-cel for follicular lymphoma, similar future comparisons will be possible [Table 1, (5)]. Taken together, histologic subtype is a possible factor, though confounding factors such as volume and extent of tumor burden, CAR-T expansion/peak, and selected agent make clear associations challenging to infer.

### Vascular risk factors

The presence of microvascular and macrovascular insults, fluid attenuated inversion recovery (FLAIR) white matter lesions, and posterior reversible (leuko)encephalopathy syndrome (PRES) observed on imaging (56, 57) suggest an underlying vascular pathology may be at play in ICANS that leads to secondary central nervous system (CNS) injury after CRS and/or CAR-T expansion. Consistently reported endothelial risk factor for ICANS in adults are the agonist/antagonist pair of soluble vascular growth factors known as angiopoietin-1 (Ang-1) and angiopoietin-2 (Ang-2) supports this observation. Both Ang-1 and Ang-2 act on the tyrosine kinase receptor, Tie-2. Ang-1 stabilizes endothelium on binding to Tie-2, including closing inter-endothelial gaps (58). In contrast, high levels of Ang-2 inhibits Tie-2 activity resulting in increased endothelial permeability [(59); for review Saharinen et al. (60)]. Three studies which have examined levels of Ang-1, Ang-2, and the ratio of Ang-2/Ang-1 in axi-cel (38) and the investigational CD19-directed agents JCAR014 (28) and 19-28z (32) with generally congruent findings: Faramand et al. found baseline (pre-lymphodepletion) serum levels of Ang-2 (*p* = 0.0190) and Ang-2/Ang-1 ratio (*p* = 0.0056) were elevated in patients who developed grade 3+ neurotoxicity (F57). Among post-infusion factors, an elevated peak Ang-2/Ang-1 ratio (*p* = 0.0016) and depressed nadir Ang-1 level (*p* = 0.0298) were also associated with grade 3+ neurotoxicity (38). Gust and colleagues found a trend to increased baseline serum Ang-2/Ang-1 ratio (*p* = 0.09) and significant post-infusion peak Ang-2 (*p* = 0.0003), and Ang-2/Ang-1 ratio (*p* = 0.0014) elevations in patients with grade 4+ neurotoxicity, although no difference in nadir post-infusion Ang-1 was observed (*p* = 0.13) (28). Finally, Santomasso and colleagues also observed significant post-infusion day 7 Ang-2/Ang-1 ratio elevation (*p* = 0.0310), but not either day 7 Ang-2 elevation (*p* = 0.07) or day 7 Ang-1 depression (*p* = 0.053) in patients with grade 3+ neurotoxicity (32).

While the pre-treatment Ang-2/Ang-1 ratio may be a promising predictive biomarker for higher grade ICANS, there remain several caveats warranting examination. First, similar findings were not observed in pediatric populations receiving CD19-directed CAR-T agents, suggesting possible differences in pediatric vs. adult susceptibility factors (36). Second, other more non-specific plasma markers such as drops in platelet count and/or fibrinogen have had more varied success (Table 2). Third, data is limited for non-CD19 CAR-T cell agents. Finally, while suggestive of a link for vascular injury and subsequent ICANS risk, the mechanism remains unclear including the relationship of ICANS to blood brain barrier breakdown and systemic cytokine, myeloid, and/or CAR-T Infiltration into the CNS. Overall, Ang-1, Ang-2, and the ratio of Ang-2/Ang-1 remain a promising, if under-explored, risk factor for ICANS.

### Neurologic comorbidities and injury

History of neurologic comorbidities or injury is another commonly reported risk factor for neurotoxicity (Table 4). Neurologic comorbidities may reflect prior CNS injury (e.g., seizures), peripheral nervous system (PNS) injury (e.g., neuropathy), CNS involvement of cancer (e.g., CNS lymphoma), or neurotoxic treatment exposure (e.g., CNS radiation, intrathecal methotrexate). Unfortunately, the definition of neurologic comorbidities varied significantly between studies. Gust and colleagues used an inclusive definition and found univariate (*p* = 0.006) and multivariate association (*p* = 0.002) when further controlling for tumor burden, increased CAR-T expansion, lymphodepletion regimen, and infused CAR-T dose in adults (28). A small recent adult cohort likewise clustered different sources of injury together and reported a possible univariate association (44). Gofshteyn and colleagues separately examined history of brain radiation, seizures, AED use, and pre-existing deficits in a pediatric cohort. Only pre-existing deficits (*p* = 0.01) was associated with encephalopathy, seizure, and aphasia, all key hallmarks of ICANS (31). Another small pediatric cohort observed 3 of 5 patients with history of pre-existing neurologic deficits developed ICANS (33). However, an association is not always observed in pediatric cohorts; Gust et al. did not observe a relationship when clustering different neurologic comorbidities except an abnormal MRI prior to treatment in a pediatric cohort (36). Overall, categorically-defined neurologic injury remains a possible risk factor, particularly in adults, though the underlying localization of injury remains unclear.

**Table 4:** Summary of studies examining baseline neurologic injury divided by variable.

	**Study**	**Agent**	**Study Population**	**Measurement**	**Relationship**
**Categorical variable**	Gust et al. (28)	Investigational (JCAR014)	Adult B-ALL, NHL, CLL	Preexisting neurologic comorbidities (peripheral neuropathy, CNS involvement, headache, intracranial hemorrhage, seizures, cognitive impairment, methotrexate toxicity, other)	Uni- & Multivariate association along with tumor burden, increased CAR-T expansion, lymphodepletion regimen, infused CAR-T dose
	Gofshteyn et al. (31)	Tasi-cel	Pediatric ALL	Pre-existing neurologic deficit, NOT history of seizures or brain radiation	Univariate association between pre-existing neurologic deficit and neurotoxicity
	Shalabi et al. (33)	Investigational (Anti-CD22)	Pediatric B cell malignancy	Pre-existing neurologic deficit	3 of 5 patients w History of pre-existing neurologic deficits developed ICANS
	Möhn et al. (44)	Tasi-cel	Pre-existing neurologic conditions	History of neurological conditions (e.g. epilepsy or history of headache, toxicity after previous methotrexate therapy)	Possible univariate association reported
	Gust et al. (36)	Investigational (SCRI-CAR19v1)	Pediatric ALL	Preexisting neurologic comorbidities (peripheral neuropathy, CNS involvement, Abnormal prior MRI, headache, intracranial hemorrhage, seizures, cognitive impairment, methotrexate toxicity, PRES)	Abnormal prior MRI but NOT other preexisting neurologic comorbidities
**Continuous variable**	Schoeberl et al. (45)	Axi-cel, Tasi-cel, investigational	Adult lymphoma	Serum NfL	Increased at baseline and further after symptom onset (as a cohort)
	Butt et al. (46)	Axi-cel, Tasi-cel, investigational	Adult lymphoma	Plasma NfL	Increased at baseline and throughout treatment up to 30 days; no further elevation after symptom onset
	Gust et al. (49)	Investigational (multiple)	Pediatric leukemia or lymphoma	Serum NfL serum GFAP	NOT baseline NfL, GFAP

Agent(s) and study population are included for reference. ALL, Acute lymphoblastic leukemia; B-ALL, B-cell acute lymphoblastic leukemia; CLL, Chronic lymphocytic leukemia; CNS, Central nervous system; GFAP, Glial fibrillary acidic protein; NfL, neurofilament light chain; NHL, Non-Hogkin's lymphoma; PRES, Posterior reversible encephalopathy syndrome.

More recently neurofilament light chain (NfL) has been used to provide quantitative, rather than categorical, assessment of neural injury (Table 4). NfL is a structural component of long axons whose levels in cerebral spinal fluide (CSF) and blood increase after a neurological insult. Baseline elevations in NfL have been reported in two adult cohorts (45, 46), with elevations predating lymphodepletion. Levels further remain elevated throughout the course of treatment for up to 30 days after infusion (46). A similar relationship is not observed in a pediatric cohort, where global elevations were observed across all patients independent of ICANS risk (49). The disagreement between the adult (45, 46) and pediatric studies (49) may reflect different assay sensitivities or previous treatment dosing/regimens. Furthermore, like vascular injury markers listed above, the mechanistic link in ICANS between neuroaxonal injury, vascular injury, blood brain barrier breakdown, and CNS cytokine, myeloid, and/or CAR-T cross infiltration in ICANS remains unclear. While promising, further study is needed to account for possible confounding factors including neuropathy and systemic vascular injury unrelated to the blood brain barrier.

## Cellular therapy factors

We previously discussed CD19-directed therapies, particularly those containing a CD28 costimulatory domain, have the highest incidence of ICANS (Table 1). This section summarizes additional cellular therapy-related risk factors known to be associated with neurotoxicity when controlling for the agent type. Additional risk factors include type/dose of lymphodepletion condition regimen, CAR T dose and expansion, are reviewed as follows:

### Lymphodepletion

Cyclophosphamide and fludarabine containing regimens are commonly used for lymphodepletion prior to CAR-T cell infusion. The dose intensity of cyclophosphamide and addition of fludarabine are associated with improved CAR-T kinetics, expansion, and response in both pediatric and adult patients treated with CD19-directed agents (24–26, 35). Early studies demonstrated an association between the lymphodepletion regimen and ICANS in both univariate and multi-variate modeling (24, 25, 28). Notably all three of these studies all examined the same experimental agent, JCAR014. Unfortunately this relationship is not universally seen across CD19-directed agents and was not observed for investigational agents, including SCRI-CAR19v1 (26, 36) or 19–28 z (30, 35). Indeed a recent retrospective study of 152 patients treated with tisa-cel also revealed no association between low (termed “suboptimal” <13.8 mgxh/L) fludarabine exposure and ICANS (61). This variability may stem from a secondary relationship with neurotoxicity, where better lymphodepletion results in improved expansion and peak CAR-T levels, which in turn is associated with neurotoxicity. Fludarabine exposure is also known to cause a rare but separate neurotoxicity from ICANS, as was observed in the ZUMA-3 trial (10). Therefore, in select cases, misdiagnosis of neurotoxicity related to fludarabine may be another source of variability that confounds the clinical assessment for ICANS. Rates of ICANS in bendamustine and other fludarabine-free lymphodepletion regimes warrant further investigation. Overall, standardization of lymphodepletion regimens and monitoring/consideration for Flu-associated neurotoxicity remains key for risk modulation.

### Dose of CAR T and expansion

Higher doses of infused CAR-T cells has been associated with increased risk for CRS and neurotoxicity, particularly in patients with high tumor burden [Tisa-cel: (42, 44); JCAR014: (24, 25, 28)]. This association was not observed for other CD19-directed agents such as the investigational 19–28 z (30, 35) or SCRI-CAR19v1 (26, 36). Overall CAR-T dose remains an unclear risk factor, particularly given the standardization of target dosing per patient weight.

In contrast to dose, the degree of expansion remains a consistent risk for the development of ICANS across multiple studies and agents (Table 2). Following infusion, CAR-T cells redistribute and proliferate rapidly, with the early expansion coupled to cytokine production and CRS risk. The duration of expansion varies, with CAR-T targeting leukemia and multiple myeloma associated with a slightly longer duration than those targeting lymphoma [for detailed comparison of kinetics, see Liu et al. (62)]. The association between expansion and ICANS risk was first observed in the original trials leading to approval of axi-cel (6, 7, 10), brexu-cel (9), liso-cel (A69), and several investigational CAR-T products including JCAR014 (24, 25, 28), 19–28 z (30, 32), SCRI-CAR19v1 (36). Of note 41 who examined 19–28 z in a pediatric cohort did not observe a relationship between neurotoxicity and expansion (*p* = 0.14), though this may reflect a small sample size (*n* = 25). Overall, rate of expansion and maximum CAR-T expansion capability remains a key risk factor for the development of neurotoxicity.

## Inflammatory factors

Development and severity of CRS remains a key risk factor for ICANS (Table 2). Consorted effort has been devoted to understand the temporal cascade of systemic and when available, CSF inflammatory cascade after treatment with a given agent (21, 63). Here we highlight the two of the most commonly reported marker in each class using a standardized grouping before focusing in particular on pre-treatment risk factors.

### Pro-(inflammatory) activation markers: IL-1 and IL-6

Post-treatment peak IL-6 elevations remain tightly coupled with the development of ICANS across multiple prospective and retrospective cohorts (Table 2). This includes the seminal trials leading the to the approval of axi-cel (6, 7, 10) and brexu-cel (9), but interestingly not tisa-cel (45). Elevations in IL-6 are a known hallmark of CRS. Given the high degree of association between CRS and ICANS, further correlation between IL-6 and ICANS is unsurprising though it remains unclear which level of contribution they provide to ICANS independently and specifically.

Along with IL-6, the IL-1 signaling pathway is considered a key mediator in CRS. Preclinical models (intraperitoneal Raji tumor cells injected SCID-beige subsequently treated with human 1928 z CAR T cells) demonstrated a tight coupling between the IL-1 signaling cascade and severe CRS through macrophage/monocyte activity (64). Of the two major agonist IL-1 ligands IL-1α and IL-1β, IL-1β is the predominately macrophage and monocyte activator with secondary effects on non-immune cells. Currently, the role of IL-1 and associated monocyte activation beyond CRS in neurotoxicity remains under active investigation. In preclinical studies, SGM3 mice treated with human CD19 CAR T cells demonstrated seizures and paralysis may be abrogated through IL-1 but not IL-6 blockade (65). This has led to active clinical investigation of IL-1 receptor antagonism using anakinra to intervene on the development of CRS and ICANS.

### Pro-proliferation markers: IL-15 and GM-CSF

Along with IL-6, IL-15, which promotes T and NK cell proliferation and activation, has been observed to be consistently elevated in individuals who develop ICANS (6, 7, 25, 28–33). The myeloid proliferation and activation cytokine GM-CSF is likewise a commonly observed blood marker whose peak levels after treatment are associated with ICANS in several retrospective and prospective studies (6, 7, 9, 30, 32, 33).

### Immuno-modulators: IL-10 and IFN-γ

Both IL-10 and IFN-γ are immune-modulators, with IL-10 traditionally thought of as an immunosuppressive regulator that decreases cytokine production and antigen presentation by antigen-presenting cells (66–68). However, high levels of IL-10 have been associated with IFN-γ-mediated CD8(+) T cell cytotoxicity (68, 69). Given known marked elevations in acute ICANS of IFN-γ, this second pathway paradoxical coupling to IFN-γ elevations is possible mechanism warranting further study.

### Baseline systemic factors

A subset of studies have examined baseline blood markers in 19–28 z (32) and axi-cel (7, 10, 38, 46). Santomasso et al. found baseline elevations in IL-6, IL-10, GM-CSF, and G-CSF, but not ferritin, in patients treated with 19–28 z who went on to develop ICANS. Faramand et al. (38) also observed baseline elevations in IL-6 in patients treated with Axi-cel who went on to develop ICANS. In contrast to Santomasso et al., baseline ferritin elevations were observed in this study and in more recent studies (7, 38, 46). Overall, baseline IL-6 and ferritin elevations are a possible risk factor, though data is limited beyond a subset of CD19-directed agents.

## Implications and future directions

Predictive biomarkers hold promise for the early, reliable, and rapid identification of patients most at risk for ICANS, despite ongoing limitations. While the mechanism underlying ICANS remains unclear, this review highlights the tight association between ICANS risk and key inflammatory, cellular therapy, and host factors in patients undergoing cellular therapy (Figure 1). Rubin and colleagues demonstrated the promise of a combined risk model for ICANS that assembles markers across each of the three major categories (39). Amidi et al. (70) recently expanded on this approach. Notably, neither study integrated vascular factors, neurologic injury, key cytokines, or type of cellular therapy agent in their respective models, holding promise these further additions may improve generalizability. Ongoing multi-omic approaches to identify new biomarkers (71), efforts to pool multi-center data as validation cohorts, and advances in preclinical mechanistic studies hold promise for a validated model that hones in on key pathologic pathways in the near future.

## Author contributions

OB: study concept, visualization, and writing—original draft. AZ: writing—original draft. BA: project administration, supervision, and critical review. JD: supervision and critical review. AG: study concept, project administration, supervision, and critical review. All authors contributed to the article and approved the submitted version.
